# Tet-mediated DNA methylation dynamics affect chromosome organization

**DOI:** 10.1093/nar/gkae054

**Published:** 2024-02-01

**Authors:** Hao Tian, Pengfei Luan, Yaping Liu, Guoqiang Li

**Affiliations:** Biomedical Pioneering Innovation Center (BIOPIC), Beijing Advanced Innovation Center for Genomics, Peking University, Beijing 100871, China; Department of Medical Genetics, Capital Institute of Pediatrics, Beijing 100020, China; Division of Human Genetics, Cincinnati Children's Hospital Medical Center, Cincinnati, OH 45229, USA; Department of Pediatrics, University of Cincinnati College of Medicine, Cincinnati, OH 45229, USA; Biomedical Pioneering Innovation Center (BIOPIC), Beijing Advanced Innovation Center for Genomics, Peking University, Beijing 100871, China

## Abstract

DNA Methylation is a significant epigenetic modification that can modulate chromosome states, but its role in orchestrating chromosome organization has not been well elucidated. Here we systematically assessed the effects of DNA Methylation on chromosome organization with a multi-omics strategy to capture DNA Methylation and high-order chromosome interaction simultaneously on mouse embryonic stem cells with DNA methylation dioxygenase Tet triple knock-out (Tet-TKO). Globally, upon Tet-TKO, we observed weakened compartmentalization, corresponding to decreased methylation differences between CpG island (CGI) rich and poor domains. Tet-TKO could also induce hypermethylation for the CTCF binding peaks in TAD boundaries and chromatin loop anchors. Accordingly, CTCF peak generally weakened upon Tet-TKO, which results in weakened TAD structure and depletion of long-range chromatin loops. Genes that lost enhancer–promoter looping upon Tet-TKO showed DNA hypermethylation in their gene bodies, which may compensate for the disruption of gene expression. We also observed distinct effects of Tet1 and Tet2 on chromatin organization and increased DNA methylation correlation on spatially interacted fragments upon Tet inactivation. Our work showed the broad effects of Tet inactivation and DNA methylation dynamics on chromosome organization.

## Graphical Abstract

**Graphical Abstract:**
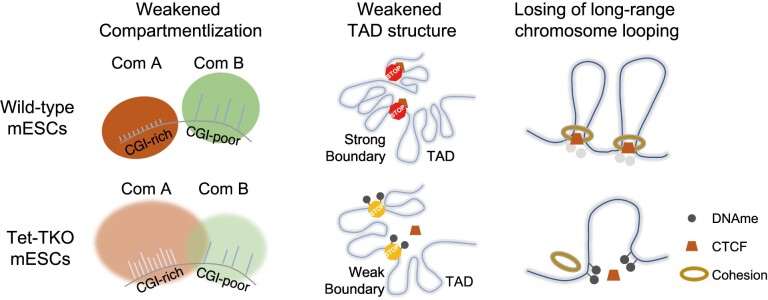

## Introduction

The eukaryotic genome is modulated by a variety of epigenetic modifications such as DNA methylation and histone modifications and must be folded to store in the nucleus. With the development of chromosome conformation capture technologies, especially genome-wide capture technologies such as Hi-C (1), ChIA-PET (2) and Micro-C (3), the general principle of high-order genome architecture has been uncovered. Briefly, the chromatin fiber can form chromatin looping to physically contact spatially proximal but linearly distal loci such as enhancers and promoters. The chromatin looping and contacts are generally restricted into insulated, million-base-sized topologically associating domains (TADs), which are highly conserved among different cell types (4,5). The chromatin loop anchors and TAD boundaries are enriched with CCCTC-binding factor (CTCF) occupancy. Disruption of CTCF binding on loop anchors and TAD boundaries could result in disrupted enhancer–promoter interactions and consequently the dysregulation of specific genes in certain conditions (6–9). Above TADs, the chromosomes are compartmentalized into active and inactive compartments (1) corresponding to euchromatin and heterochromatin, respectively, and the switch of compartment often contributes to the activation or repression of specific genes. Although the cohesin-mediated loop extrusion model provides a molecular basis for chromosome organization, the underlying mechanisms and regulators for the dynamics of chromosome organization in a variety of biological processes such as cell differentiation (10) are still limited. Therefore, a detailed dissecting of the modulators for genome organization is needed.

DNA methylation has been shown to affect chromatin states by negatively correlating with chromatin accessibility (11,12) and facilitating chromatin condensation in specific regions (13). Previous work has shown that DNA methylation nadirs that have large genomic intervals and exhibit low DNA methylation levels could facilitate the formation of CTCF-independent long-range chromatin interactions (14), suggesting the possible involvement of DNA methylation in guiding genome architecture. In fact, many studies have already investigated the effects of knock-out (KO) of DNA methylation methyltransferases (DNMT1, DNMT3a, and DNMT3b), which induce the global hypomethylation, on high-order genome architecture. For instance, researchers have shown that CTCF binding remained stable upon the loss of DNA methylation by triple KO of DNMT1, DNMT3a, and DNMT3b, and the TAD insulation was not affected (15,16) while others found a decreased compartment strength in both DNMTs double knock-out cells (17) and 5-AZA treated cells (18). On the other hand, DNA hypermethylation can affect specific chromosome interactions by blocking the occupancy of CTCF in chromatin loop anchors and TAD boundaries, which has been illustrated by the classic example of allelic chromatin loops mediated H19-IGF2 imprinting status through allelic DNA methylation blocking of CTCF binding (19,20). DNA hypermethylation could also reduce CTCF binding, resulting in unexpected spatial interactions between enhancer and oncogene and consequently the oncogenic expression (21). However, a comprehensive analysis of to what extent the DNA hypermethylation will affect 3D chromatin organization including compartment, TAD insulation, and chromatin looping is still lacking.

DNA methylation is dynamic and properly regulated in normal conditions and the genome-wide dysregulation of DNA methylation is a hallmark of human cancers (22). The 5-methylcytosine (5mC) modification can be reversed by both passive DNA demethylation during DNA replication and active DNA demethylation by Tet-mediated oxidation of 5-methylcytosine (5mC) to 5-hydroxymethylcytosine (5hmC) (23). 5hmC is enriched at distal regulatory elements such as enhancers, H3K4me1-marked regions, DNase hypersensitivity sites, and transcription-factor-bound regions. Similarly, knocking out of Tet in mouse embryonic stem cells mainly affected the methylation status of distal elements (24) rather than genome-wide DNA methylation changes compared to DNMTs KO (17,25). While these studies investigated the effect of DNA hypermethylation upon Tet KO on activities of distal elements like enhancers, previous works have shown that DNA hypermethylation was largely confined to the euchromatins while DNA hypomethylation was also observed in heterochromatin in TET-deficient genome (26), suggesting that Tet-mediated DNA methylation change is complex. Although recent work also showed that Tet inactivation could disrupt YY1 binding (27), the broad effects of Tet inactivation on chromatin interactions between these *cis*-regulatory elements and their targeting genes as well as the chromosome organization have not been investigated.

Here, we examined the effects of the loss of Tet dioxygenases on chromosome organization and DNA methylation. By generating genome-wide DNA methylation and chromosome contacts via multi-omics Methyl-HiC that simultaneously captures the DNA methylation and chromosome interaction (28) on mouse embryonic stem cells with Tet triple knock-out (Tet-TKO), we elucidated that DNA methylation variation mediated by Tet oxidation can affect chromosome interactions and organization. Consistent with the previous study (24), we observed enriched DNA hypermethylation in enhancers from Methyl-HiC data. We found globally weakened chromosome compartmentalization in Tet-TKO cells that corresponds to decreased methylation differences between CGI-rich and CGI-poor domains. We also observed significantly reduced chromatin loops and weakened TAD structure upon Tet-TKO that may result from the weakened CTCF and YY1 peaks. We further dissected the knocking-out effect of individual Tet dioxygenases on genome architecture and found Tet1 and Tet2 have distinct impacts on chromatin architecture. With the unique feature of Methyl-HiC to capture DNA methylation and chromatin interactions at the single-molecular level, we found Tet-TKO could enhance DNA methylation concordance in interacting reads. With these new analyses, our work provided new insights regarding the crosstalk between DNA methylation and 3D genome organization.

## Materials and methods

### Cell culture

The mouse embryonic stem cell line E14 and its Tet-TKO were gifts from Yi Zhang's lab at Harvard Medical School as previously described (24). The J1 wild-type, Tet1-KO, and Tet2-KO cells were from Chuan He's lab at the University of Chicago. mESCs were cultured on 0.2% gelatin-coated feeder-free dishes in a medium with 85% DMEM, 15% Knock-out Serum Replacement (Gibco, 10828–028), 1X penicillin/streptomycin, 1X non-essential amino acids (Gibco, 11140-050), 1X GlutaMax (Gibco, 35050), 0.4 mM β-mercaptoethanol and 1000 U ml^−1^ LIF (Millipore, ESG1107). Cells were passed every two days to maintain proper condensation and pluripotency.

### Methyl-HiC experiment

Methyl-HiC was performed as previously described (28). Briefly, 2 million cells were cross-linked with 1% formaldehyde for 10 min at room temperature. The reaction was then quenched with 0.2 M Glycine. Cell pellets were washed with cold PBS and lysed with lysis buffer to get nuclei pellets. Nuclei were permeabilized with 0.5% SDS. DNA was *in situ* digested with 100 units of DpnII (NEB, R0543) overnight. The ends of restriction fragments were filled with biotinylated nucleotides (Invitrogen, 19524016) and *in situ* ligated with T4 DNA ligase (NEB, M0202). After the reversal of cross-links, ligated DNA was ethanol precipitated and sheared to about 400 base pairs (bp) by sonication (Covaris). Sonicated products were pulled down with streptavidin beads to enrich interacted fragments. After adapter ligation, beads were suspended in 20 μl TE buffer and subjected to bisulfite conversion with EZ DNA Methylation-Gold Kit (Zymo, D5005). Unmethylated lambda DNA (Promega, D1501) was sonicated and ligated with the same adapter for the Methyl-HiC sample and then was spiked in at 0.5% before bisulfite conversion. Purified bisulfite-converted DNA was amplified with HiFi Hotstart Uracil + Ready Mix (KAPA, KK2802). The libraries were sequenced on Illumina HiSeq 2500 or HiSeq 4000 sequencer.

### *In situ* Hi-C experiment

Cells were processed in the same way as for methyl-HiC before the bisulfite conversion steps. Briefly, after the adapter ligation step, the DNA fragments were directly amplified with PCR on beads. The supernatant was purified by 0.7× Ampure XP beads, quantified, and submitted for paired-end sequencing on Illumina HiSeq 2500 or HiSeq 4000 sequencer.

### ChIPmentation experiment

ChIPmentation experiments of CTCF on E14 WT and Tet-TKO were performed as previously described (29) with a few modifications. Briefly, cells were fixed with 1% formaldehyde at room temperature for 10 min followed by glycine quenching. Nuclei were isolated and chromatin was fragmented to 200–700 bp using Covaris (S220). Antibody (anti-CTCF: Abcam, ab128873) was added to the sheared chromatin, and complexes were incubated on a rotator o/n at 4°C. Blocked Dynabeads (Thermo Fisher Scientific, 11204D) were then mixed with antibody/chromatin complexes, all of which were incubated together at 4°C for 2h rotating. The beads/antibody/chromatin complexes were then subjected to ChIPmentation by incubating with homemade Tn5 transposase in tagmentation reaction buffer for 10 min at 37°C. The pulldown DNA was recovered by reversing crosslink overnight followed by SPRISelect beads (Beckman Coulter Life Sciences, B23318) purification. ChIP-seq libraries were prepared using the NEBNext® High-Fidelity 2× PCR Master Mix (New England Biolabs, M0541S) with illumina nextera primers according to the manufacturer's instructions. Size selection was then done with SPRISelect beads to choose the fragments ranging from 100 to 1000 bp.

### Methyl-HiC and Hi-C data process

Methyl-HiC data were processed with Bhmem as previously described (28). Based on the bam file, bam2pairs was then used to generate contact files. Short-range contacts with genomic distance less than 1 kb were removed and the final .hic format files were generated using juicer_tools (30,31) (v1.22.01). Genome-wide DNA methylation data were also extracted from the bam file using MethylDackel (v0.5.1, https://github.com/dpryan79/MethylDackel) ‘extract’ function with ‘mergeContext’ with minimal coverage above 10 reads. Besides, perRead function in MethylDackel (v0.5.1) was used to obtain DNA methylation information at the read level. Hi-C data were processed using HiC-Pro (v3.1.0) (32).

### Downstream analysis of contact matrices

Chromatin loops, as well as differential chromatin loops, were identified using Mustache (33) at 10 kb (E14 WT VS E14 Tet-TKO) and 25 kb resolution (8-cell versus ICM, HCT116 versus DKO1). TADs were identified at 50 kb resolution using hicFindTADs in HiCExplorer (34–36) (v3.0) with minDepth (200 000), maxDepth (500 000), step (50 000) and thresholdComparisons (0.05). Compartments were identified at 250 kb resolution using ‘compartments’ in FAN-C (37) (v0.9.24) and GC contents were utilized to decide the compartment type.

ATA (aggregate TAD analysis), APA (aggregate peak analysis), inter- and intra-TAD interaction calculation, and compartment enrichment analysis were achieved using ‘ATA’, ‘APA’, ‘intra_inter_TAD’, and ‘saddle’ functions in GENOVA (38) (v1.0.0.9000), respectively, which scaled the genome-wide sum of reads to 1e9 for direct comparison among different samples. DMR aggregation analysis was also achieved using PESCAn function in GENOVA (38) (v1.0.0.9000). We performed chromatin loop annotation with R package GenomicInteractions (39) (v1.29.5), with enhancer and promoter coordinates obtained from chromHMM data (strong enhancer) and FANTOM5 project (https://fantom.gsc.riken.jp/5/), respectively.

### DMR identification and annotation

DMRs were identified using DSS (40) (v2.40.0), with regions covering at least 5 CpGs, methylation difference above 20%, and p.threshold below 0.01, respectively. DMR annotation was achieved with package ChIPseeker (41) (v1.28.3).

### ChIPmentation data process

Raw fastq data were trimmed using trim_galore (v0.6.6, https://github.com/FelixKrueger/TrimGalore) and then mapped to the mm9 genome reference using bowtie2 (42) (v2.4.4). The deduplication process was achieved by the markdup function in samtools (43) (v1.12). Finally, the bamCoverage function in deepTools (44) (v3.5.1) with CPM normalization was used to generate the final bigwig files for further downstream analysis.

### Enrichment analysis

CTCF peaks were identified using MACS2 (v2.2.7.1) (45). The distributions of CTCF, Tet1, DNA methylation, 5hmC, etc. around specific locations (such as CTCF peaks) were calculated using computeMatrix function in deepTools (44) (v3.5.1). Besides, genome-wide DNA methylation distribution and the corresponding difference between the two samples were calculated with multiBigwigSummary function in deepTools (44) (v3.5.1). CpG density distribution calculation was based on the mouse reference genome (mm9) using 1-kb non-overlapped windows.

## Results

### Tet triple knocking-out weakens chromatin compartmentalization by erasing domain separation

**Figure 1.**
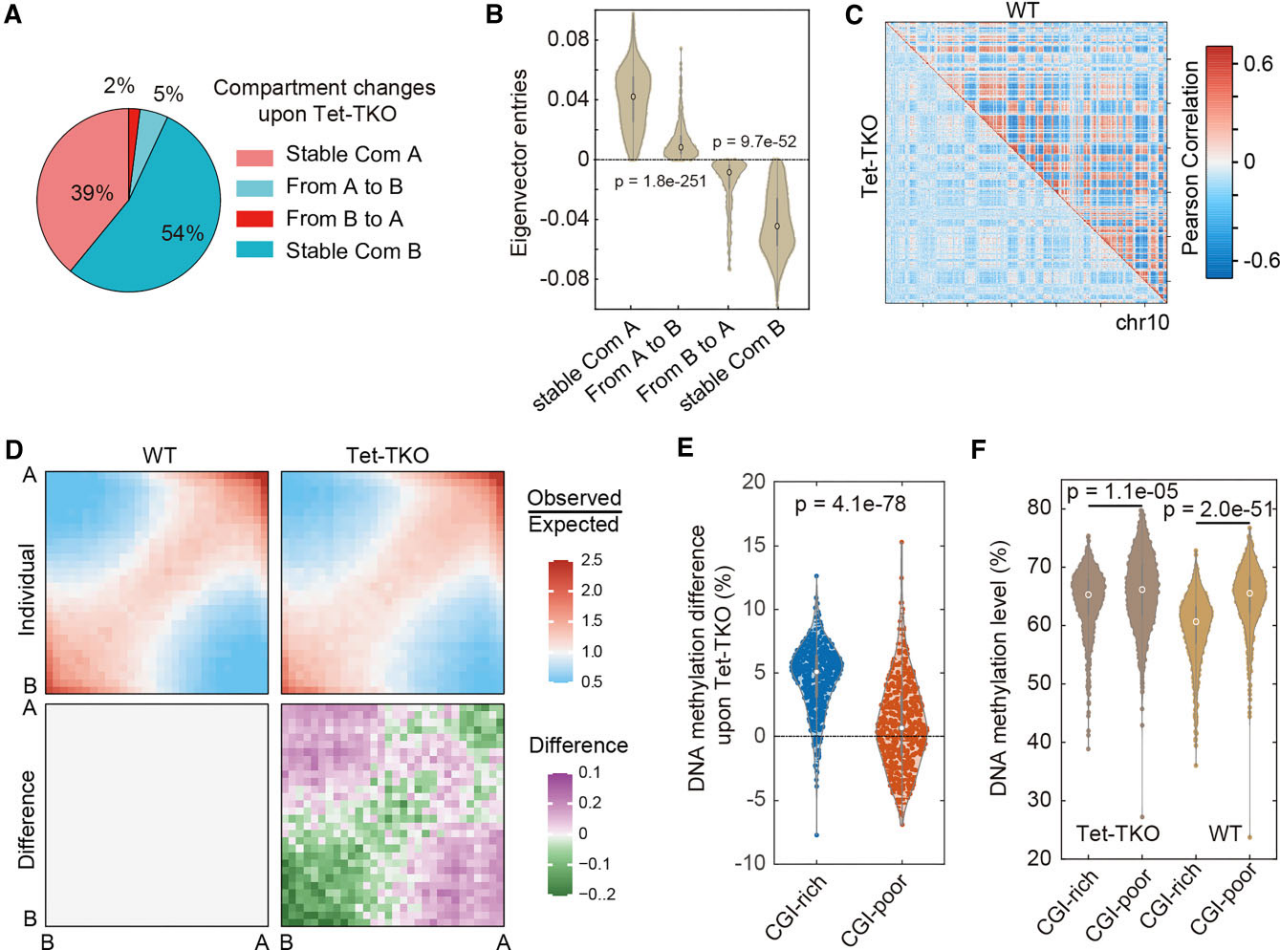
(**A**) The proportion of stable and dynamic compartments upon Tet-TKO. (**B**) Eigenvector entries (in WT) of four kinds of regions including A to B, stable A, B to A and stable B. Welch's unequal variance t-test was performed. (**C**) Pearson correlation heatmap derived from Methyl-HiC data of WT and Tet-TKO. Chr10 was used here. (**D**) Saddle plot, based on Hi-C data, revealing the compartment strength variation upon Tet-TKO. (**E**) Methylation variation of CGI-rich and CGI-poor domains upon Tet-TKO. Welch's unequal variance t-test was performed. (**F**) Methylation level of CGI-rich and CGI-poor domains in WT and Tet-TKO. Welch's unequal variance t-test was performed.

To examine the effects of DNA methylation on chromosome organization, we captured genome-wide DNA methylation and chromosome conformation with the multi-omics method Methyl-HiC as well as the classical *in situ* Hi-C for chromosome organization on mouse embryonic stem cells with Tet triple knock-out (Tet-TKO) and individual knock-outs (Tet1-KO, Tet2-KO). Our data showed comparable quality features between the two methods in raw reads numbers, *cis* interaction proportions, and contact matrix (Supplementary Figures S1A, S1B and S1C). Globally, Tet-TKO induced hypermethylation slightly (Supplementary Figure S1D), which was different from the global hypomethylation induced by Dnmt-TKO (Supplementary Figure S1E). Based on the contact matrices obtained from Methyl-HiC, we first performed compartment analysis with FAN-C (37) and used GC content to assign compartments A and B, which show higher active histone modification and Dnase signals but lower DNA methylation levels in compartment A compared to compartment B (Supplementary Figures S1F and S1G). In general, in line with a previous study (26), we found that compartments A and B tend to be hyper- and hypo-methylated, respectively (Supplementary Figure S1H), with a significant positive correlation between eigenvector entries and methylation variation observed for both compartments A and B (Supplementary Figure S1I), hinting the possible link between 1-D and 3-D information. We then compared the distribution of compartments between WT and Tet-TKO cells and found that the majority of compartments remained stable with 5% switching from A to B and 2% from B to A, respectively (Figure 1A). Compared to stable compartments, those switched compartments tend to have lower absolute eigenvector entries (Figure 1B). Moreover, regions switching from compartment A to compartment B are characterized by lower Dnase and H3K4me3 signals in WT cells, suggesting that less open and active regions in compartment A are more vulnerable to Tet triple knocking-out (Supplementary Figure S1J).

Although the distribution of compartments A and B was generally stable, previous studies observed a significant decrease of compartmentalization strength after global loss of DNA methylation in both DNMT-DKO cells and 5-AZA treated cells (17,18). To check whether opposite compartmentalization changes could be observed in Tet-TKO cells, we plotted the Pearson correlation heatmap derived from Methyl-HiC contact matrices (Figure 1C) and found the absolute PCC (Pearson correlation coefficient) significantly decreased upon Tet-TKO, indicating a weakened compartmentalization. We also performed compartment enrichment analysis in contact matrix generated from both Methyl-HiC and *in situ* Hi-C datasets and observed that interactions within the same compartments (A–A and B–B) decreased whereas interactions across different compartments (A–B) increased (Figure 1D and S1K). Taken together, these results show that compartmentalization is weakened upon Tet triple knocking out.

Previous works have shown that the CpG density distribution is strongly associated with the chromatin organization, particularly the nuclear space could be phase-separated according to CpG island (CGI) enrichment into CGI-rich (CGI forest) or CGI-poor (CGI prairie) domains that correspond to compartments A and B, respectively (46,47). Notably, the DNA methylation difference between CGI-rich and CGI-poor correlates very well with the strength of domain segregation or compartmentalization, suggesting that the degree of the methylation difference between CGI-rich and CGI-poor domains may influence compartment strength (47,48). We, therefore, examined whether weakened compartmentalization correlated with decreased DNA methylation difference between CGI-rich and CGI-poor domains. Interestingly, both Tet-TKO and Dnmt-TKO resulted in decreased DNA methylation differences between CGI-rich and CGI-poor domains (Figures 1E, F, S1L and S1M), although through opposite directions (Figures 1E and S1L). In detail, in Tet-TKO cells, the methylation difference between CGI-rich and CGI-poor domains strikingly decreased due to the more severe hypermethylation of CGI-rich but not CGI-poor (Figure 1E). In Dnmt-TKO cells, both CGI-rich and CGI-poor domains underwent significant hypomethylation but a more severe extent was observed for CGI-poor domains (Supplementary Figure S1M), which also resulted in the decreased methylation difference between domains. In summary, both Dnmt- and Tet-TKO could decrease the DNA methylation difference between CGI-rich and CGI-poor domains, which resulted in weakened compartmentalization.

### Tet triple knocking-out affects CTCF peak binding and weakens the TAD structure

**Figure 2.**
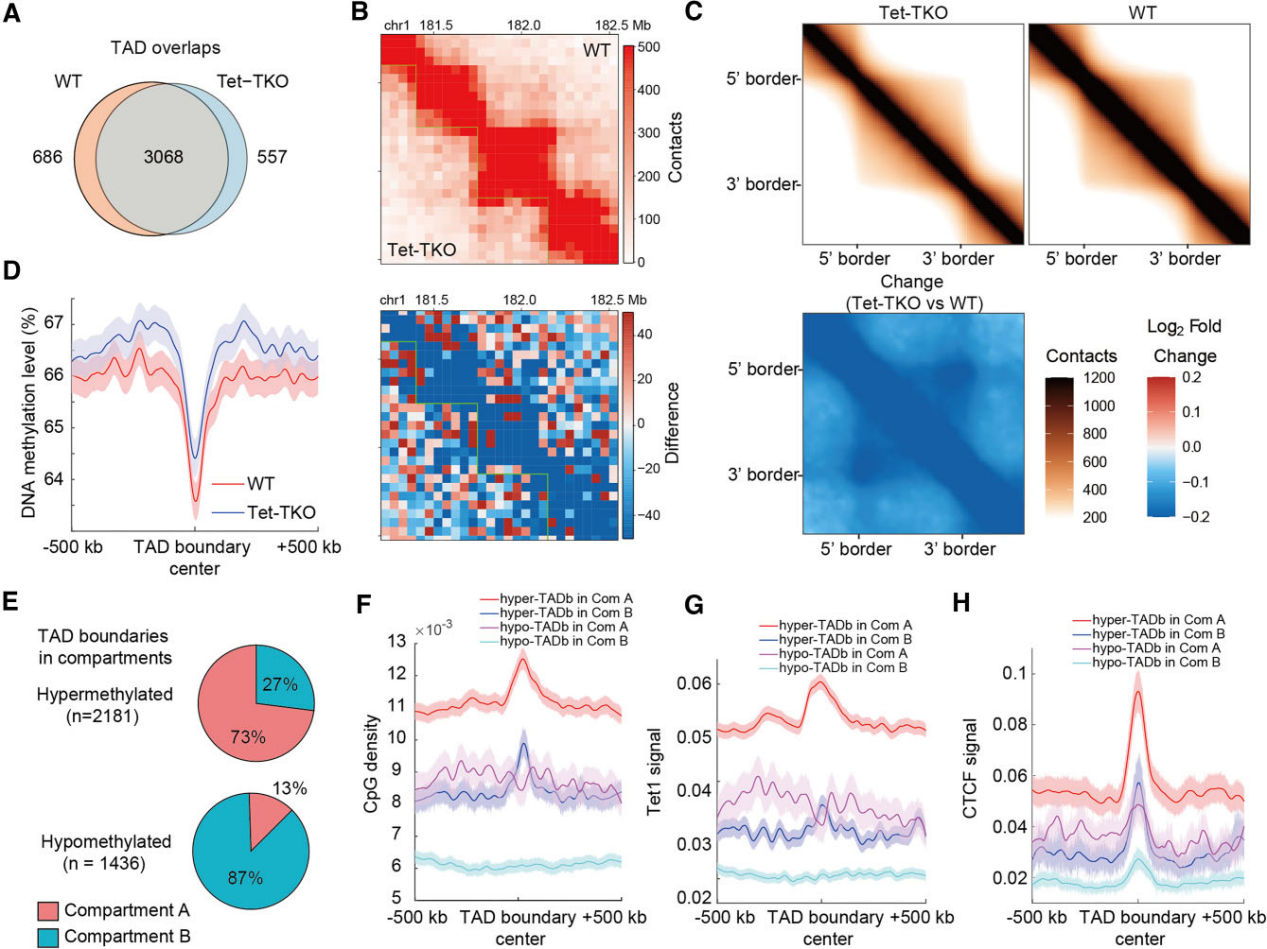
(**A**) The overlap of TADs identified in WT and Tet-TKO. Overlapped TADs were defined as the proportion of overlapped regions larger than 60% in both conditions. (**B**) An example (chr1: 181–182.5 Mb) showing intra-TAD interaction variations. (**C**) ATA (aggregate TAD analysis) revealing the TAD structure variation upon Tet-TKO. Methyl-HiC data were used here. (**D**) DNA methylation distribution around TAD boundaries in WT and Tet-TKO. (**E**) The distribution of hyper- and hypo-methylated TAD boundaries in compartments A and B. (F–H) The distribution of CpG density (**F**), Tet1 signal (**G**) and CTCF signal (**H**) around different categories of TAD boundary. Errorbar: mean ± se.

Next, we investigated the TAD structure variation upon Tet-TKO. Using HiCExplorer (34–36), we identified 3754 and 3625 TADs from the Methyl-HiC dataset in WT and Tet-TKO cells (Supplementary Table S1), respectively. While the TAD domains are generally overlapped in these two conditions (Figure 2A), we observed decreased interactions within TADs upon Tet-TKO based on aggregate TAD analysis (ATA) (Figures 2B and C) that could be further validated by *in situ* Hi-C data (Supplementary Figure S2A). We also observed more decreases in intra-TAD interactions versus inter-TAD interactions between two adjacent TADs (Supplementary Figure S2B), implying weakened TAD structure in Tet-TKO cells.

To examine whether DNA methylation changes upon Tet-TKO were associated with weakened TAD structure, we plotted DNA methylation changes around TAD boundaries and found a general hypermethylation trend in the center and surrounding regions of TAD boundaries (Figure 2D), with 5hmC and Tet1 enrichments in boundaries in wild-type cells (Supplementary Figures S2C and S2D). Using the DNA methylation profiles in Methyl-HiC data, we identified 2556 differentially methylated regions (DMRs, see Supplementary Table S2) in Tet-TKO cells with 2554 of them hypermethylated, accordant with a recent study revealing the pervasive hypermethylation upon Tet-TKO (49). The DMRs were enriched with 5hmC and Tet1 binding in wild-type cells (Supplementary Figures S2E and S2F) and a significant number of them were located in distal-regulatory elements like enhancers (Supplementary Figure S2G), which showed severe hypermethylation in Tet-TKO cells (Supplementary Figure S2H), in line with previous works with WGBS data showed that the DMRs upon Tet inactivation were highly enriched in distal regulatory elements (24).

Although we found that the DMRs were enriched in TAD boundaries (Supplementary Figure S2I) and the majority of the TAD boundaries (*n* = 2181) were hypermethylated upon Tet-TKO, we also observed a significant number of hypomethylated TAD boundaries (*n* = 1436). Furthermore, hypermethylated TAD boundaries were mainly distributed in compartment A, while hypomethylated TAD boundaries were mainly distributed in compartment B (Figures 2E), which is associated with the above observations in compartmentalization (Supplementary Figure S1H). We thus divided TAD boundaries into four categories (Figure 2E) and examined their sequence and chromatin mark properties. Regions surrounding TAD boundaries in compartment A always tend to be hypermethylated regardless of the TAD boundary behaviors (Supplementary Figure S2J, left two panels), in contrast, regions adjacent to TAD boundaries in compartment B are not hypermethylated (Supplementary Figure S2J, right two panels). Besides, hypermethylated boundaries are characterized by the peak of CpG density distribution in the TAD boundary (Figure 2F). Intriguingly, further inspection revealed that the distribution of CTCF and Tet1 around the TAD boundary (in WT) indeed resembled that of CpG density (Figures 2G and H, the CTCF and Tet1 data were downloaded from the available database (see Supplementary Table S5), indicating the close association between sequence and chromatin mark. Generally, in WT, as for the regions up- and downstream of TAD boundaries, compartments A possess higher CpG density, CTCF, and Tet1 signals, compared to compartments B expectedly. By contrast, within TAD boundaries, hypermethylated TAD boundaries harbored higher signals. Taken together, the more open environment in WT for hypermethylated TAD boundaries may facilitate the Tet1 binding, letting the hypermethylated TAD boundaries be more susceptive upon Tet-TKO and finally resulting in the hypermethylation behavior.

**Figure 3.**
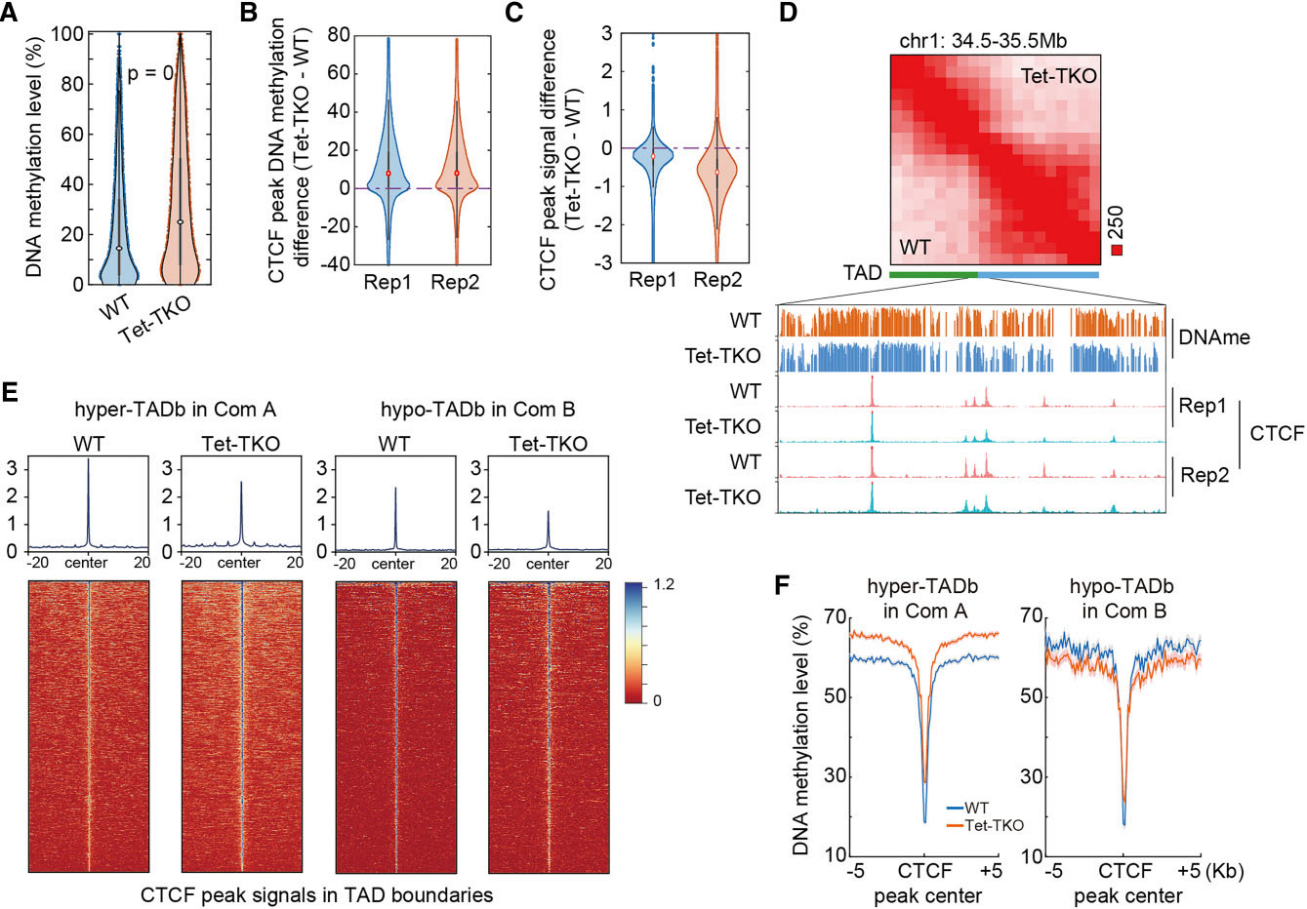
(**A**) DNA methylation level of CTCF peaks in TAD boundary. T-test was performed. (**B**) DNA methylation difference of CTCF peaks upon Tet-TKO for two replicates. (**C**) CTCF peak signal variations upon Tet-TKO for two replicates. (**D**) An example (chr1: 34.5–35.5 Mb) showing the CTCF and DNA methylation distributions around one TAD boundary. (E, F) The CTCF signal (**E**) and DNA methylation (**F**) distribution around CTCF peaks residing in hypermethylated TADb in compartment A and hypomethylated TADb in compartment B.

We next focus on the CTCF binding changes upon Tet-TKO to investigate whether CTCF binding variation could be connected to TAD structure variation. To this end, we performed CTCF ChIPmentation experiments with two biological replicates (29). Firstly, we found that the CTCF peaks were overall hypermethylated in both compartments upon Tet-TKO (Figures 3A, B and S3A), which may hinder CTCF binding as previous works suggest (21). The results indeed showed that upon Tet-TKO, although CTCF peaks are generally conserved (Supplementary Figure S3B), the CTCF binding strength on these peaks was globally weakened (Figures 3C, D and S3C–F). Interestingly, we found that the CTCF peaks became hypermethylated and less bounded by CTCF in both hypermethylated and hypomethylated TAD boundaries (Figures 3E and F). Taken together, these results suggested that the hypermethylation of CTCF binding peaks in TAD boundaries may impede CTCF binding, resulting in weakened CTCF peak, further contributing to weakened TAD structure.

### Losing of long-distance chromatin loops upon Tet-TKO

**Figure 4.**
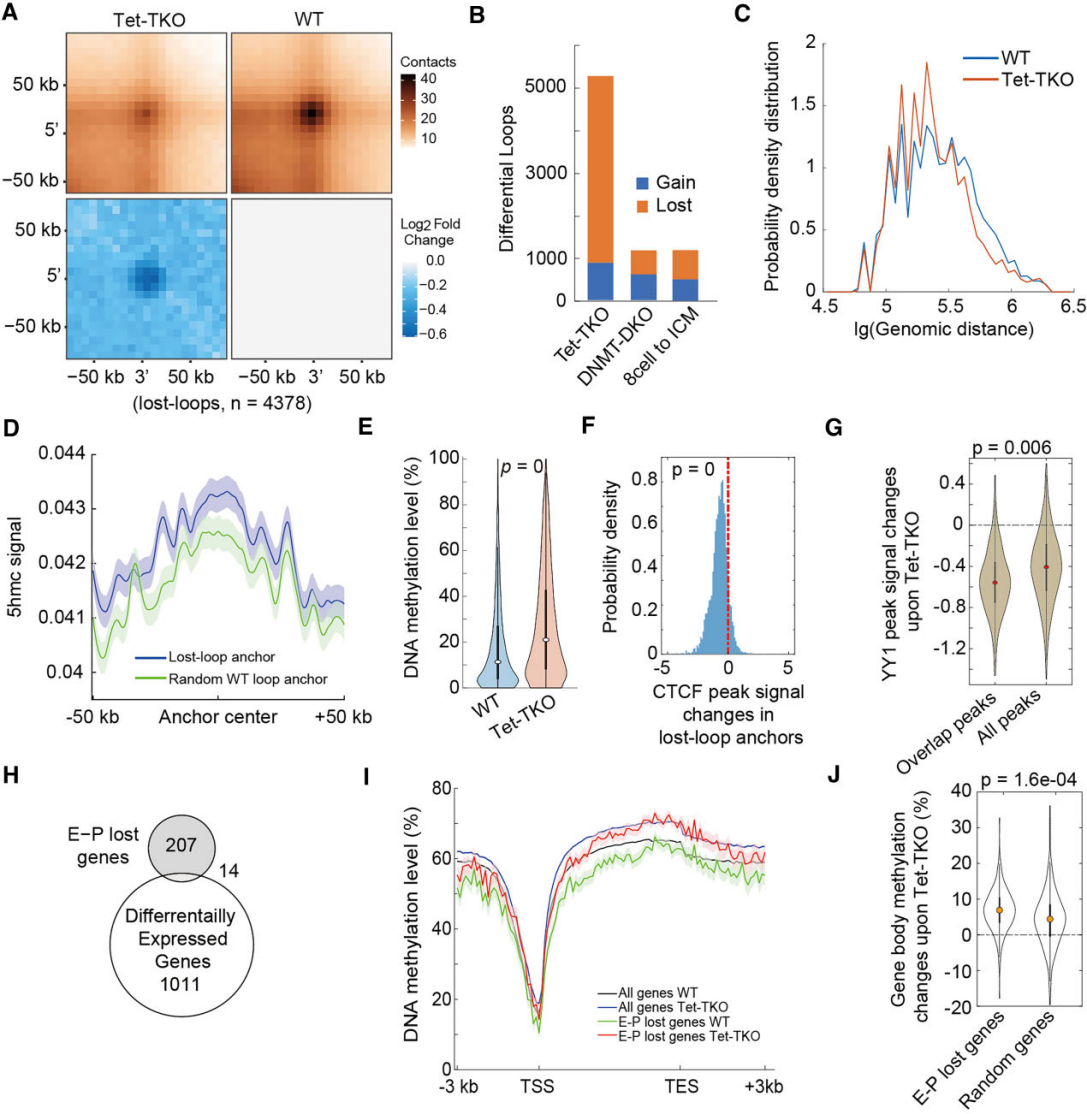
(**A**) APA (aggregation peak analysis) for Lost-loops (*n* = 4378). (**B**) Comparison of the number of differential loops between several conditions. (**C**) The distribution of chromatin loop size in WT and Tet-TKO. Methyl-HiC data were used here. P-value = 4.1e-15 by Welch's unequal variance *t*-test. (**D**) 5hmc signal around lost-loop anchors and randomly selected WT loop anchors. (**E**) DNA methylation level of CTCF peaks residing in lost-loop anchors. T-test was performed. (**F**) The variations of signals of CTCF peak in lost-loop anchors. T-test was performed. (**G**) YY1 peak signal variations upon Tet-TKO. ‘overlap peaks’ means YY1 peaks overlapping with Tet1 peaks. Welch's unequal variance t-test was performed. (**H**) Venn diagram revealing the overlap between differentially expression genes (DEG) and genes losing enhancer-promoter interactions. (**I**) The methylation distribution for genes losing enhancer-promoter interactions and all genes (*n* = 22 380). (**J**) Gene body methylation difference upon Tet-TKO of genes losing enhancer-promoter interactions and randomly selected genes with the same number. Welch's unequal variance t-test was performed.

Previous works have shown that Tet knocking-out mainly affects distal elements like enhancers (24,50). We were curious whether chromatin loops linked distal elements to their cognate genes were also affected upon Tet knocking-out. To address this, we used Mustache (33) to identify chromatin loops with the Methyl-HiC dataset and *in situ* Hi-C dataset, respectively. Both datasets showed significantly fewer loops in Tet-TKO cells compared to wild-type cells (Supplementary Figure S4A, Supplementary Table S3). We further identified differential loops and found a significantly higher number of lost loops (*n* = 4378) compared to gained loops (*n* = 877) in Tet-TKO cells with a loss-to-gain ratio of 5 (Figures 4A and B), which was further validated by *in situ* Hi-C data (Supplementary Figure S4B). This is very different from that of global hypomethylation (Supplementary Figure S4C) in early embryonic demethylation from 8-cell to ICM stages as well as in DNMT-DKO cells (17), both of which showed almost equally gained and lost loops (Figure 4B, Supplementary Table S4), suggesting that the global loss of chromatin loops was specific to Tet inactivation. We next categorized the chromatin loops by chromatin compartmentalization and genome elements and found that the proportions of different categories for Lost-loops were consistent with that in WT and Tet-TKO cells (Supplementary Figure S4D and S4E), indicating the global loss of long-range chromatin loops was unbiased and not region-specific. Interestingly, the genomic distance of chromatin loops in Tet-TKO was significantly shorter than those in wild-type cells, indicating that long-range interactions were more vulnerable to being disrupted in Tet-TKO cells (Figures 4C and S4F).

We then examined whether the loss of chromatin loops was also associated with hypermethylation and CTCF peak variation upon Tet-TKO. We first found that loop anchors tend to enrich 5hmC modification, and the enrichments were even higher for Lost-loop upon Tet-TKO (Figure 4D). Moreover, compared to randomly selected Lost-loops, the Lost-loops whose anchors overlapped with DMR underwent more severe genome reorganization (Supplementary Figure S4G). Similarly, the spatial interactions between DMRs were also significantly decreased (Supplementary Figure S4H). Next, we focused on the CTCF peaks within Lost-loop anchors and found that accordant with the aforementioned results, the CTCF peaks were hypermethylated and the CTCF signals in peaks became weakened (Figures 4E, F and S4I). We also examined peak signals of YY1, another key player in mediating chromatin loops, with published ChIP-seq data (27). We found that YY1 peak generally weakened upon Tet-TKO, and the extent of decrease was higher for YY1 peaks overlapping with Tet1 peaks (Figure 4G), agreeing with the previous observation that Tet inactivation could disrupt YY1 binding (27). Taken together, these results showed that upon Tet-TKO, the disruption of CTCF and YY1 binding may contribute to the loss of chromatin loops.

### Gene body hypermethylated for genes lost enhancer-promoter interactions upon Tet-TKO

Next, we checked whether the loss of chromatin loops could affect gene expression. We focused on the lost loops linking enhancers and promoters (E-P lost loops, *n* = 221). Surprisingly, we observed very limited genes with E-P lost loops were differentially expressed (Figure 4H), with 10 genes up-regulated (*Abt1*, *BC051019*, *Ebf1*, *Fgf15*, *Fzd6*, *Got1l1*, *Myo7a*, *Pcsk9*, *Sall2* and *Samd4*) and 4 genes down-regulated (*Cd2*, *D6Wsu163e*, *Gpx2*, and *Slc22a18*). This observation is consistent with multiple previous works showing the low correlation between loop structure and gene expression (51–53). Notably, for these genes losing their enhancer-promoter interactions, we observed a higher degree of hypermethylation in their gene bodies compared to the genic background and the random cases (Figures 4I, J, S4J and S4K). Many studies have revealed that gene body methylation was positively correlated with gene expression (46,54,55), mechanistically by preventing RNA pol II binding at abnormal transcription starting sites to avoid spurious transcription initiation (56,57). Therefore, we speculated that the low correlation between enhancer-promoter interaction and gene expression may be associated with the gene regulatory functions of gene body methylation and further experiments are needed.

### Distinct effects of Tet1 and Tet2 on chromosome organization

**Figure 5.**
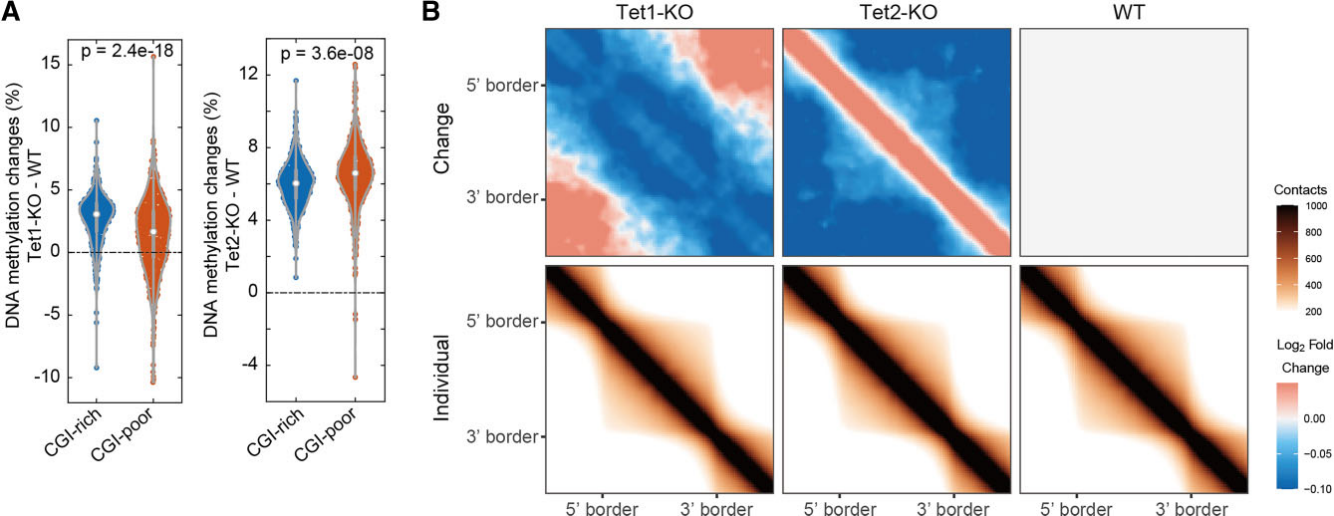
(**A**) DNA methylation variations of CGI-rich and CGI-poor domains upon Tet1-KO and Tet2-KO. Welch's unequal variance t-test was performed. (**B**) ATA analysis revealing the intra- and inter-TAD interaction variations upon Tet1-KO and Tet2-KO. Methyl-HiC data were used here.

Tet1 and Tet2 have been shown to have a distinguishable effect on both 5hmC abundance and genomic distributions, with Tet2 having a more dominant effect (50). To check whether this is also true for chromosome organization, we performed Methyl-HiC and *in situ* Hi-C experiments on WT, Tet1-KO and Tet2-KO cells separately, and did the same analysis as above. Consistent with observation in previous work (50), Tet2-KO induced more severe hypermethylation compared to Tet1-KO (Figures 5A and S5A). In TAD insulation, both ATA (Figure 5B) and quantitative analysis (Supplementary Figure S5B) revealed decreased intra-TAD and increased inter-TAD interactions upon Tet1-KO but an opposite direction for Tet2-KO in which inter-TAD interactions were decreased, suggesting that Tet2-KO has more severe effects on depleting long-range chromatin contacts. These observations were further validated by *in situ* Hi-C data (Supplementary Figure S5C and S5D). Taken together, these results revealed that Tet1 and tet2 have distinct effects on chromosome organization.

### Tet inactivation enhances DNA methylation concordance in spatially proximal fragments

**Figure 6.**
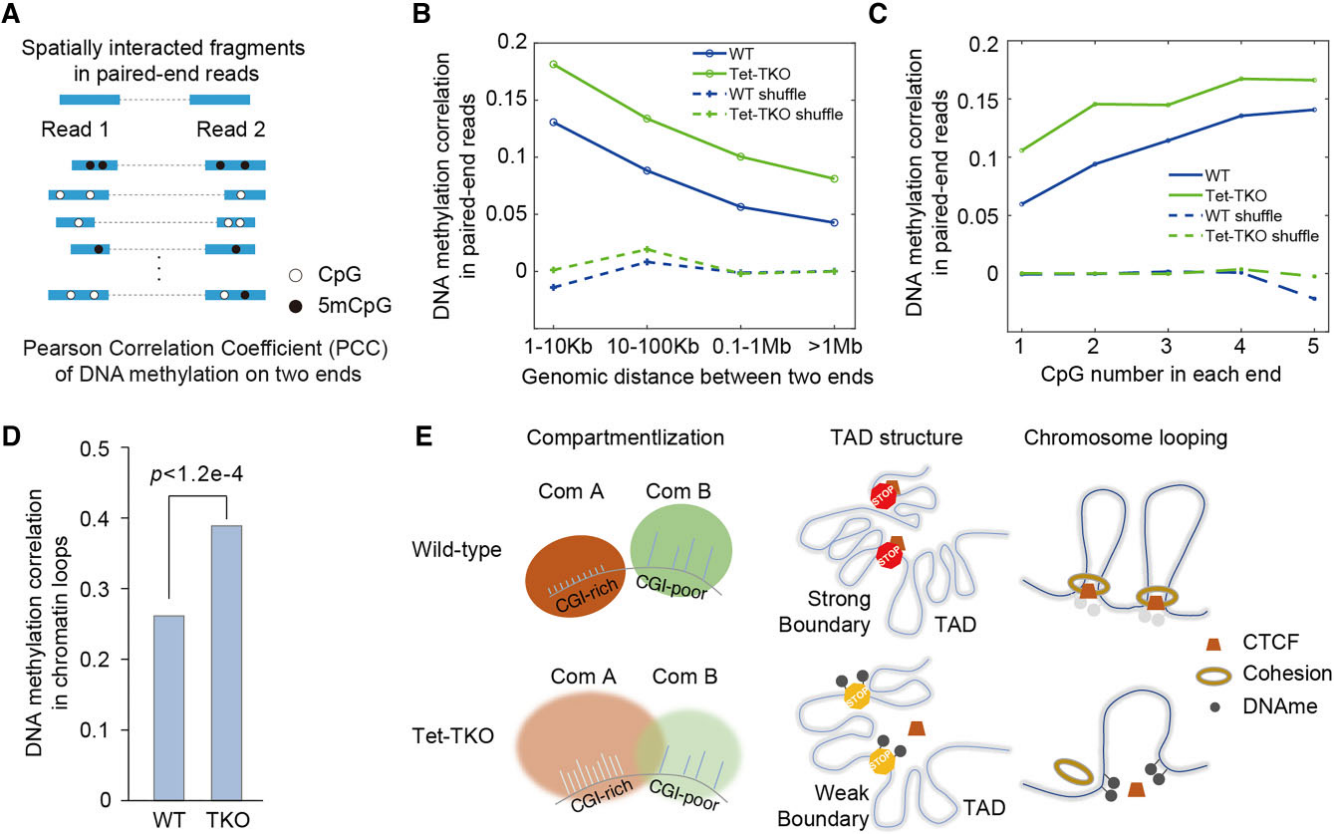
(**A**) A schematic representation showing how PCC (Pearson Correlation Coefficient) was calculated. (B, C) The association between PCC and genomic distance (**B**), between PCC and CpG number in each read (**C**). P-values for WT and Tet-TKO are all smaller than 1e-15, indicating a weak but statistically significant positive correlation. (**D**) PCC calculated on chromatin loops. (**E**) A schematic representation revealing chromatin structure variations at multiscale upon Tet-TKO.

The above results show that the inactivation of Tet could reshape chromosome organization at different levels by regulating DNA methylation status with specificity. With the uniqueness of Methyl-HiC to simultaneously capture DNA methylation and chromosome contact from the same DNA molecule, our previous work has shown that spatially proximal regions especially chromatin loop anchors tend to be concordant in DNA methylation status (28). We were curious whether Tet triple knocking out could disrupt the concordance. So, we extended the concordance analysis based on spatially interacted reads of E14 WT and corresponding Tet-TKO cells and calculated the Pearson correlation coefficient (PCC) between the DNA methylation levels in paired-end reads with distal interactions (Figure 6A). We first investigated how the genomic distance of spatial interactions could influence their DNA methylation concordance. The results showed a declined concordance with the increase of genomic distance in both wild-type and Tet-TKO cells (Figure 6B). Considering that CpG density was associated with DNA methylation and chromatin organization (46), we examined whether the number of CpGs per read possessed was related to the DNA methylation correlation coefficient and found that the correlation coefficient gradually increased with the increase of CpG numbers (Figure 6C). Notably, DNA methylation concordance in Tet-TKO cells was higher than that in WT cells spanning all different genomic distances and CpG densities (Figures 6B and C), suggesting that Tet-TKO could enhance the DNA methylation concordance in spatially interacted reads. This enhancement was also valid in the frequently interacted chromatin loops (Figure 6D). Taken together, these results suggest that Tet could add more complexities to the DNA methylation status in spatially interacted reads.

## Discussion

In this work, we performed Methyl-HiC, a multi-omics method that can simultaneously capture DNA methylation and chromosome interactions, and *in situ* Hi-C on mouse embryonic stem cells with Tet triple knock-out (Tet-TKO) and individual knock-out (Tet1/Tet2-KO). By integrative analysis, we systematically assessed the effects of Tet-TKO on chromosome organization in levels of compartmentalization, TAD insulation, and chromatin loop formation. We observed a global decrease in compartmentalization, weakened TAD structure, and a significant loss of chromatin loops upon Tet-TKO. Furthermore, we also observed distinct effects of Tet1-KO and Tet2-KO on 3D chromatin structure and DNA methylation. In the final part, we concluded that Tet inactivation could enhance DNA methylation concordance on spatially contacted fragments.

There is great interest in determining the impact of DNA methylation on the chromatin structure. A recent work by introducing DNMT expression in Saccharomyces cerevisiae has generated valuable information regarding the correlation between DNA methylation, gene expression, and chromatin organization (13). In this work, the authors observed the anti-correlation of DNA methylation with gene expression in TSS, and anti-correlation of DNA methylation with nucleosome positioning, which are conserved to that in multi-cellular organisms like humans and mice. These observations suggest that in the 1-D linear chromosome scale, DNA methylation affects chromosome states similarly in yeast and mammals. On the other end, although both Buitrago et al's work and ours revealed significant changes in chromosome conformation, the correlations of DNA methylation to 3D chromatin organization are different between yeast and mouse as we discussed in our work. In yeast, DNA methylation induces an increase in intra-chromosomic contacts while our work in mESCs revealed that hypermethylation by Tet-TKO at CTCF peak regions could impede CTCF binding, which further weakened TAD structure and result in the loss of chromatin loops. Moreover, we found the interactions between hypermethylated DMRs were also decreased. This difference may relate to the absence of chromosome architecture protein CTCF in yeast as CTCF is conserved in most bilaterian phyla but it is absent from yeast. In mammals, the plasticity of CTCF binding is linked to differential DNA methylation that could be regulated by methyltransferases and demethylation pathways such as Tet-mediated oxidation, therefore adding a more complex regulation of chromosome organization beyond the physical property of DNA methylation.

Our work with Tet inactivation also complements previous works that induce DNA demethylation via inactivation of DNMTs (17) or DNA methylation inhibitors like 5-AZA (18). In these works, the DNA demethylation also showed weakened chromatin compartmentalization as we observed in Tet triple knocking out. Chromatin compartmentalization or domain segregation is highly correlated with the CpG island (CGI) distribution in the genome, with CGI-rich domains corresponding to compartment A and CGI-poor domains corresponding to compartment B, and compartmentalization degree was suggested to be related to DNA methylation difference between these two kinds of domains (48). Here, we found that both DNMTs knock-out and Tet triple knock-out resulted in the decreased methylation difference between CGI-rich and CGI-poor domains, which implies a general mechanism of DNA methylation in regulating chromatin compartmentalization (Figure 1). In contrast to similar effects on compartmentalization between Tet-TKO and DNMTs KO, we observed distinct effects on chromatin looping exerted by Tet inactivation and DNMTs inactivation (Figure 4). We found that upon Tet-TKO, CTCF peak hypermethylation may impede the CTCF binding, resulting in the weakened CTCF peak, which may further result in weakened TAD structure and loss of chromatin loops (Figures 3 and 4), emphasizing the key roles of CTCF in mediating chromatin states and structure. In summary, we propose that in Tet-TKO cells chromatin seems to be less segregated and organized (Figure 6E). Considering that DNA methylation is highly dynamic in different cell types and Tet dioxygenases are frequently disrupted in different pathogenesis, further dissecting the association between Tet activity and chromosome organization in different cell types could help us gain more insights into the role of Tet in mediating high-order genome architecture.

Intriguingly, previous study (58) found that 5hmC could promote the R-loop formation and the depletion of Tet1, Tet2, and Tet3 significantly reduced the R-loop. Intriguingly, two back-to-back papers published in *Molecular Cell* recently showed the potential roles of R-loop in mediating 3D genome organization. Zhang et al. found that CTCF and R-loops are cohesin barriers and thus, similar to CTCF, R-loops also likely play nonnegligible roles in mediating 3D genome organization (59). Wulfridge et al. reported that G-quadruplexes, together with the R-loop, could promote CTCF binding while the attenuation of the R-loop could reduce CTCF binding (60). Considering these findings with our study, we speculate that beyond affecting chromatin organization by impeding CTCF binding directly via DNA hypermethylation, Tet inactivation may also affect chromatin organization by reducing the CTCF binding via reduction of R-loops, which may be examined in future studies.

Previous works with complete degradation of CTCF and the cohesin loading factor Nipbl have shown that active and inactive genome compartments remain properly segregated while the insulation of TADs and chromatin looping were disrupted, revealing that the formations of TADs and compartments result from distinct mechanisms: cohesin-independent compartment segregation defined by chromatin states and cohesin-dependent loop extrusion for TAD boundaries and chromatin loops (61,62). In our study, we proposed that the weakened compartmentalization (Figure 1) is due to the decreased DNA methylation difference between CGI-rich and CGI-poor domains upon Tet-TKO since the DNA methylation difference has been proved to be an indicator of domain segregation (48). On the other end, the hypermethylation of CTCF peaks may hinder the binding of CTCF therefore resulting in weakened TAD structure and loss of chromatin loops. Therefore, our work agrees with this independent model very well and provides further evidence for that.

The 3D genome field is continuously debating the effects of chromatin organization on gene expression, with many studies showing a low correlation between chromatin looping and gene regulation (51–53). For example, a recent study (63), which investigated how SARS-Cov-2 affected 3D chromatin architecture, found that for some strongly up-regulated genes, enhancer activities and spatial interactions were reduced, while the dramatic gain of H3K4me3 signals in promoters was suggested to compensate the gene expression. Gene body methylation has been widely reported to be positively correlated with gene expression (46,55,64,65). Mechanistically, gene body methylation could prevent spurious transcription initiation and retrotransposon elements, which could improve the overall gene transcription efficiency (56,57,65). Accordingly, we observed a higher gene body hypermethylation degree for those genes that lost their enhancer-promoter interactions. Therefore, based on these studies and our results, we propose that the low correlation between losing enhancer-promoter interactions and differential gene expression may be due to a compensation mechanism originating from gene body hypermethylation. Our work also suggests that further works with better examination of the chromatin states surrounding the genes is critical to interpreting the relationship between gene regulation and high-order genome organization.

## Supplementary Material

gkae054_Supplemental_Files

## Data Availability

Raw data generated in this study have been deposited to the Genome Sequencing Archive (GSA) under the accession numbers CRA012043 for Methyl-HiC and *in situ* Hi-C, CRA012010 for CTCF ChIPmentation. Previously published data used in this study have been summarized in Supplementary Table S5. Any additional information required to reanalyze the data can be obtained from the lead contact upon request.
