# Supplementary material for: Tet-mediated DNA methylation dynamics affect chromosome organization
Source: Nucleic Acids Res. 2024 Feb 1;52(7):3654–66. doi: 10.1093/nar/gkae054 (PMC11039979; doi:10.1093/nar/gkae054)
Supplement: gkae054_Supplemental_Files [file gkae054_supplemental_files.zip › Supplemental_figures.pdf]

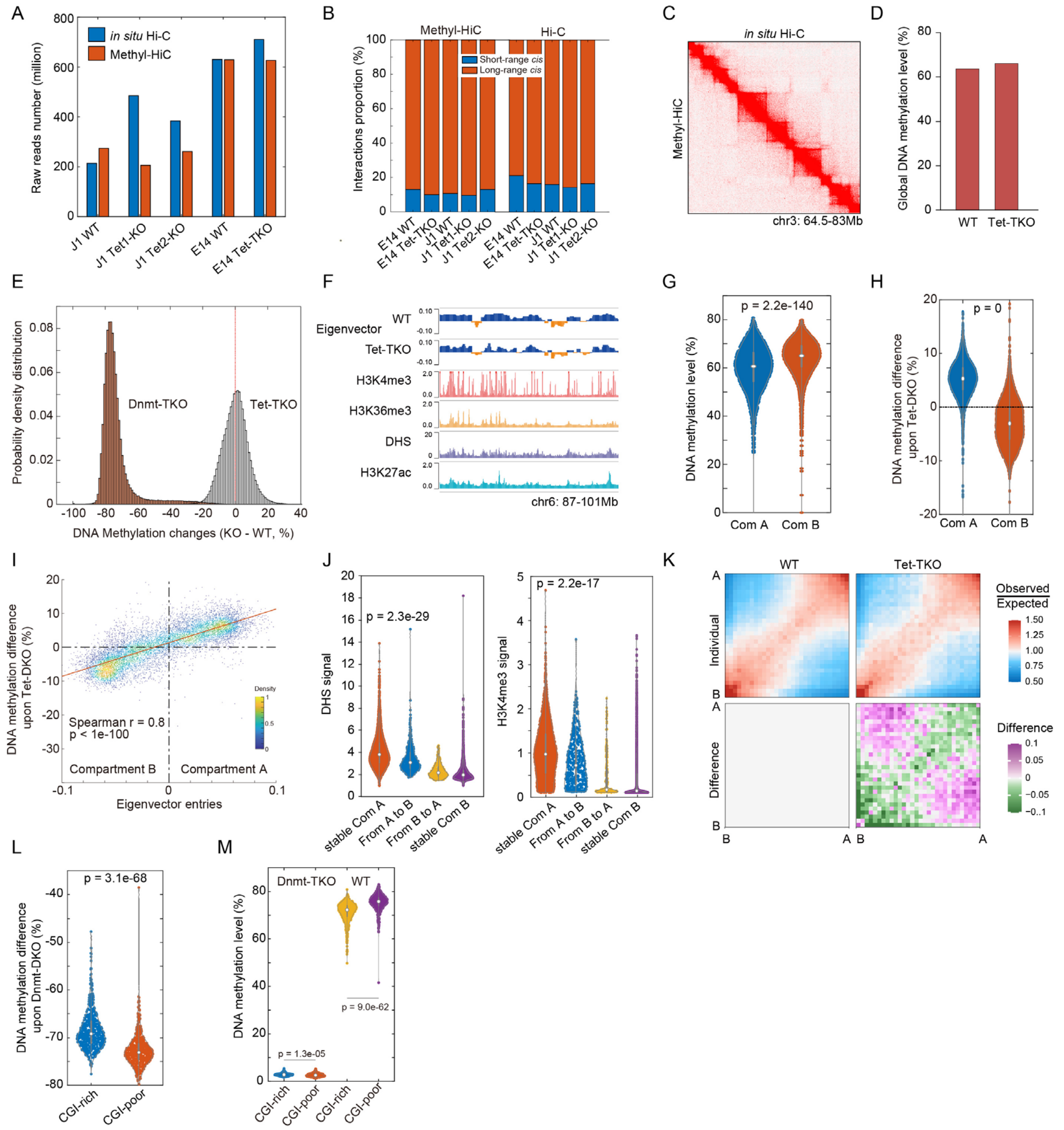

Figure S1. (**A**) The number of sequencing reads in Methyl-HiC and Hi-C experiments. (**B**) Quality control for Methyl-HiC and Hi-C data. (**C**) Comparisons of contact matrices obtained from Methyl-HiC and Hi-C experiments. (**D**) Global DNA methylation levels in WT and Tet-TKO. (**E**) The distribution of DNA methylation variations upon Tet-TKO and Dnmt-TKO. P = 0 for Welch's unequal variance t-test. (**F**) Snapshot revealing the eigenvector entries, histone modifications and Dnase. (**G**) DNA methylation level in compartments A and B. Welch's unequal variance t-test was performed. (**H**) DNA methylation variations upon Tet-TKO in compartments A and B. Welch's unequal variance t-test was performed. (**I**) The correlation between eigenvector entries and DNA methylation variations upon Tet-TKO. Spearman correlations only for compartments A and B are 0.39 ($p < 1e-100$) and 0.56 ($p < 1e-100$). (**J**) Dnase and H3K4me3 of four kinds of regions, including A to B, stable A, B to A and stable B, in WT. Welch's unequal variance t-test was performed. (**K**) Saddle

plot for compartment analysis. Methyl-HiC data were used here. (**L**) Methylation difference of CGI-rich and CGI-poor domains upon Dnmt-TKO. Welch's unequal variance t-test was performed. (**M**) DNA methylation level of CGI-rich and CGI-poor domains in WT and Dnmt-TKO. Welch's unequal variance t-test was performed.

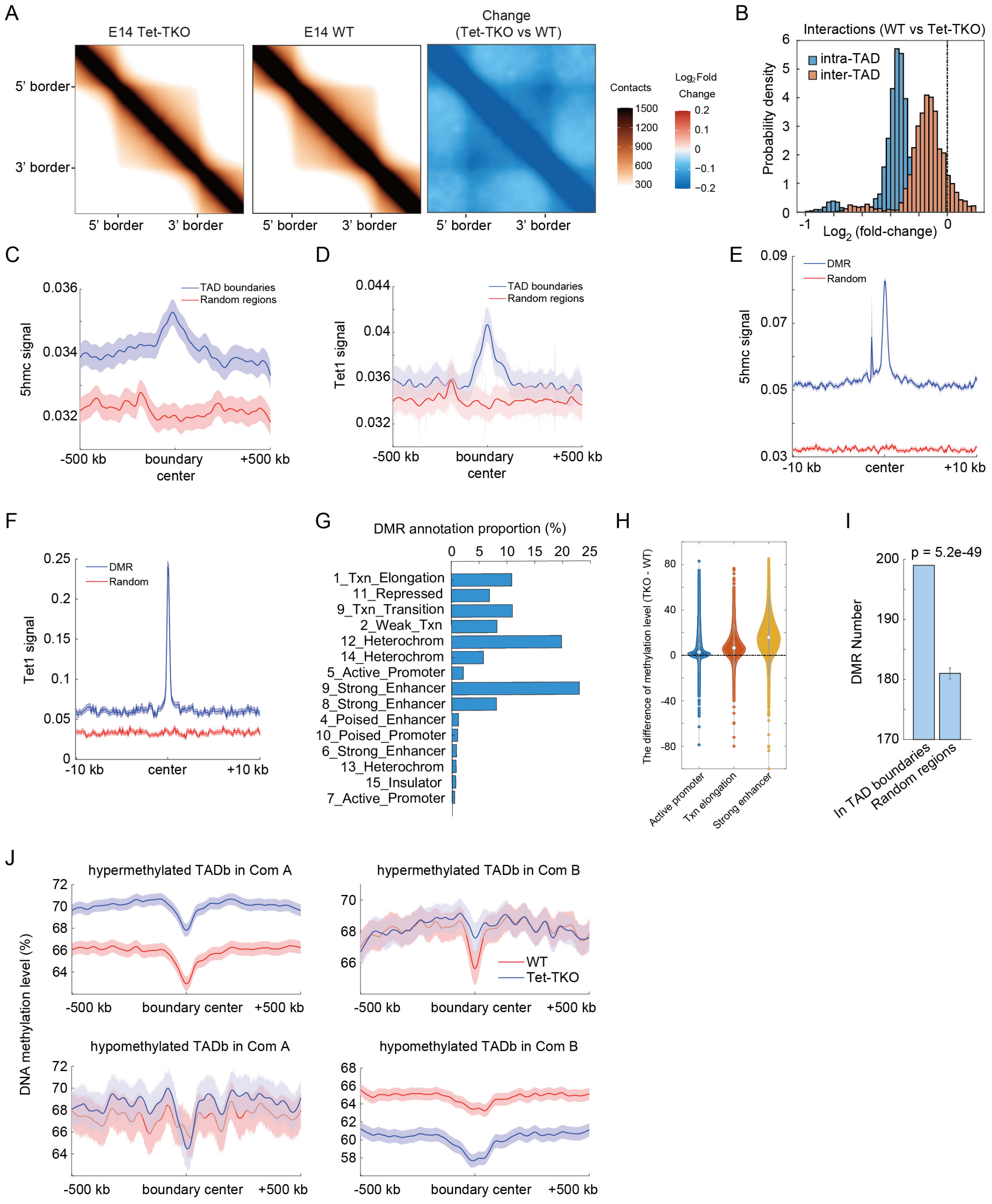

Figure S2. (**A**) ATA analysis revealing the TAD structure variation upon Tet-TKO. Hi-C data were used here. (**B**) Intra- and inter-TAD interaction (between two adjacent TADs) variations upon Tet-TKO. (**C-D**) The distribution of 5hmc (**C**) and Tet1 (**D**) around TAD boundaries. Errorbar: mean±se. (**E-F**) The distribution of 5hmc (**E**) and Tet1 (**F**) around DMR. (**G**) DMR annotation based on chromHMM data. (**H**) Methylation difference of three kinds of regions, including active promoter, transcription elongation and strong enhancer, upon Tet-TKO. P < 1e-100 for "active promoter" and "strong enhancer", and for "transcription elongation" and "strong enhancer". (**I**) The number of DMRs overlapping with TAD boundaries. For comparison, we randomly selected genome regions (using bedtools

shuffle) 200 times and calculated the number of regions located in TAD boundaries. T-test was performed. (**J**) The distribution of DNA methylation level around four kinds of TAD boundaries. Errorbar: mean±se.

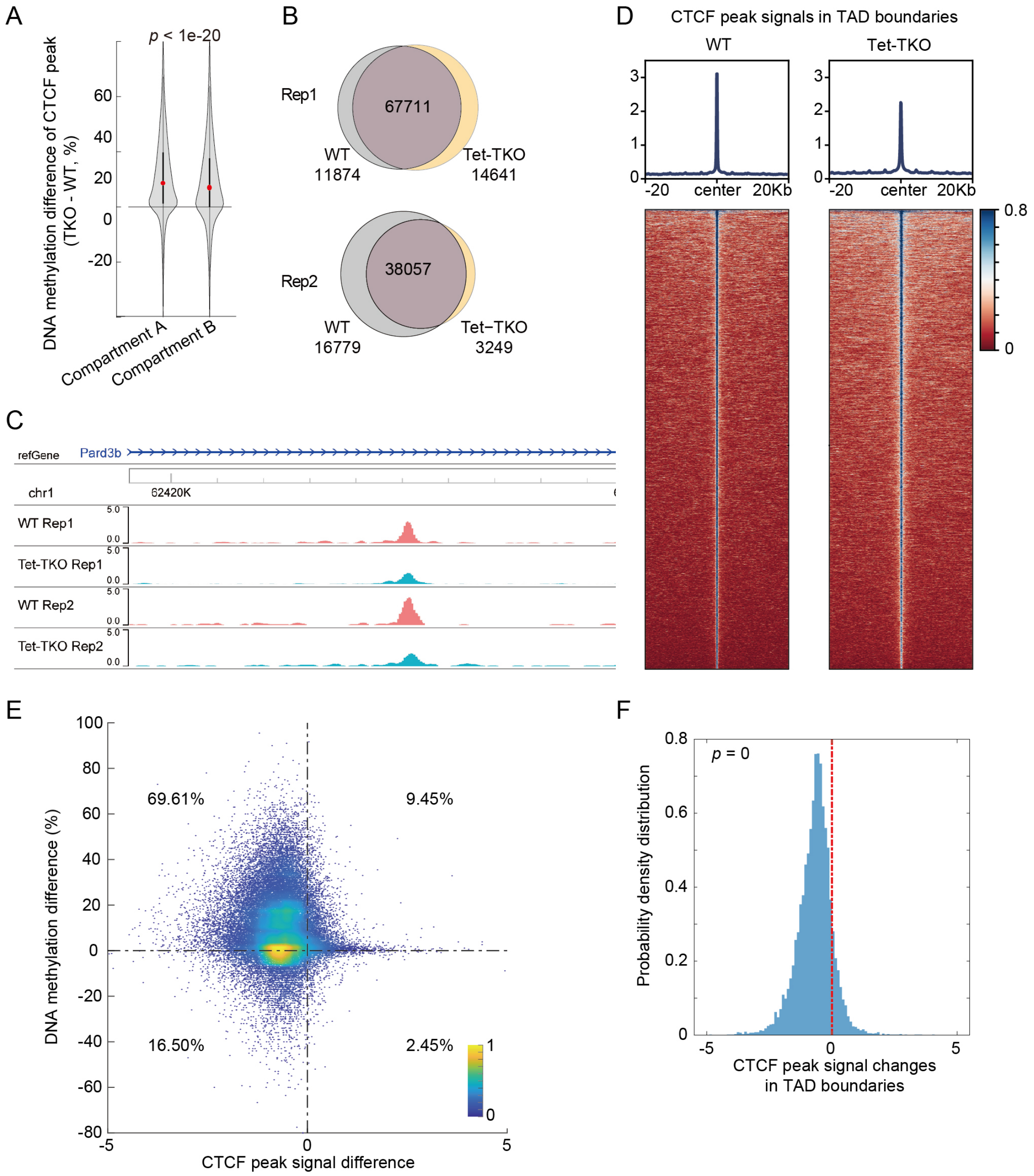

Figure S3. (**A**) DNA methylation difference of CTCF peaks in compartments A and B. Welch's unequal variance t-test was performed. (**B**) Venn diagram showing the overlap of CTCF peaks obtained from WT and Tet-TKO cells, n = 2 replicates. (**C**) An example showing weakened CTCF peak upon Tet-TKO, n = 2 replicates. (**D**) The signal distribution of CTCF peaks residing in TAD boundaries. (**E**) Scatterplot showing the variations of CTCF signal and DNA methylation (upon Tet-TKO) for CTCF peaks. (**F**) The signal variation of CTCF peaks residing in TAD boundaries. T-test was performed.

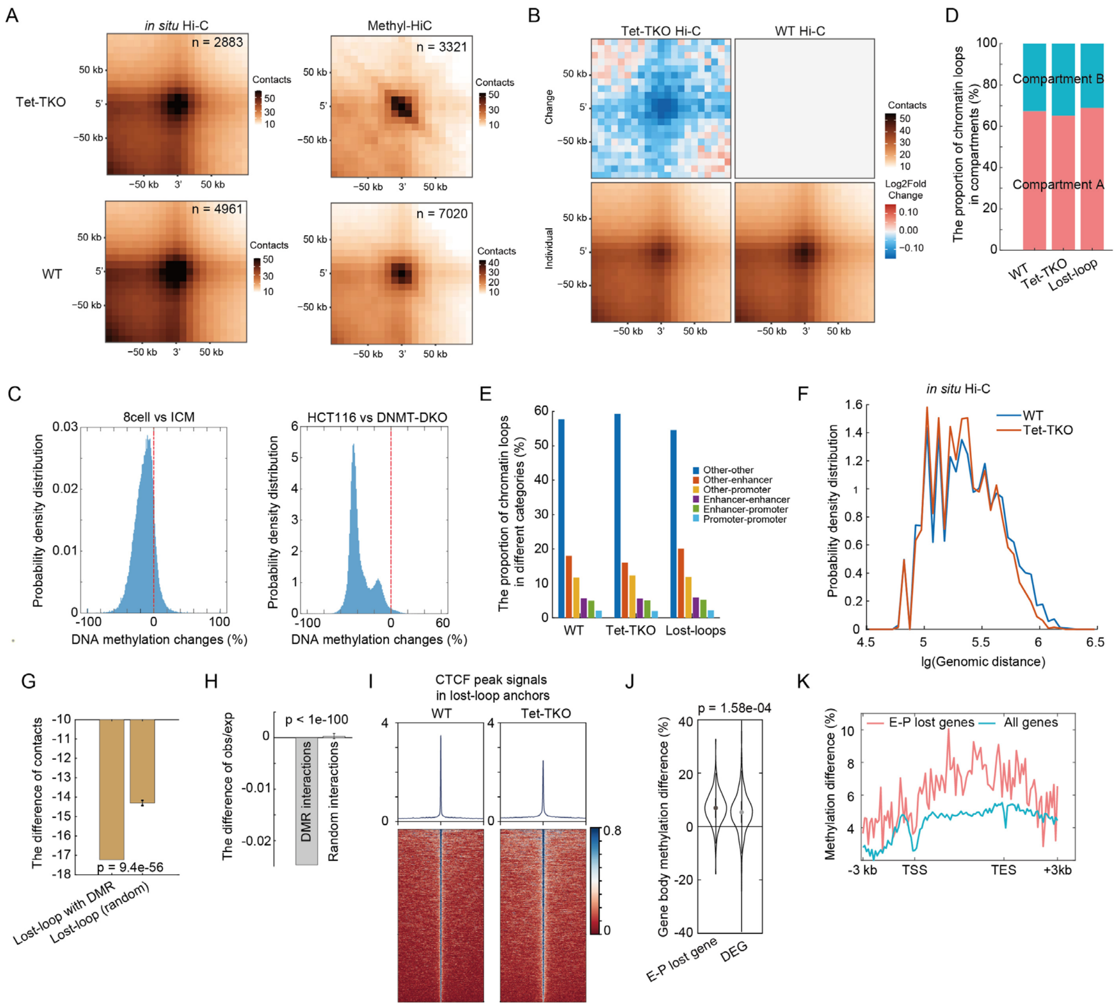

Figure S4. (**A**) APA for chromatin loops identified in WT and Tet-TKO. Left: Hi-C data, right: Methyl-HiC data. (**B**) Lost-loops identified based on Methyl-HiC are validated by Hi-C data. (**C**) Genome-wide DNA methylation difference between 8cell and ICM, and between HCT116 and corresponding DKO1 (double knock out of *DNMT1* and *DNMT3B*). (**D**) The proportions of chromatin loops in compartments A and B. (**E**) Loop categories for WT loop, Tet-TKO loop and lost-loop. (**F**) The distribution of chromatin loop size in WT and Tet-TKO. Hi-C data was used here for validation. P = 2.5e-07 for Welch's unequal variance t-test. (**G**) The contact probability variations (upon Tet-TKO) for Lost-loops whose anchors overlapped with DMR. For comparison, we randomly selected the same number of chromatin loops within Lost-loops 100 times and t-test was performed. (**H**) The obs/exp variations (upon Tet-TKO) for DMR interactions, once again, we randomly selected genome regions 100 times for comparison. P-value was calculated using t-test. (**I**) The distribution of CTCF signals around CTCF peaks residing in lost-loop anchors. (**J**) Gene body methylation variation upon Tet-TKO for E-P lost genes and DEG (differentially expressed gene). Welch's unequal variance t-test was performed. (**K**) Methylation difference upon Tet-TKO for genes losing enhancer-promoter interactions and all genes (n

= 22380). This figure is calculated based on Figure **4l**.

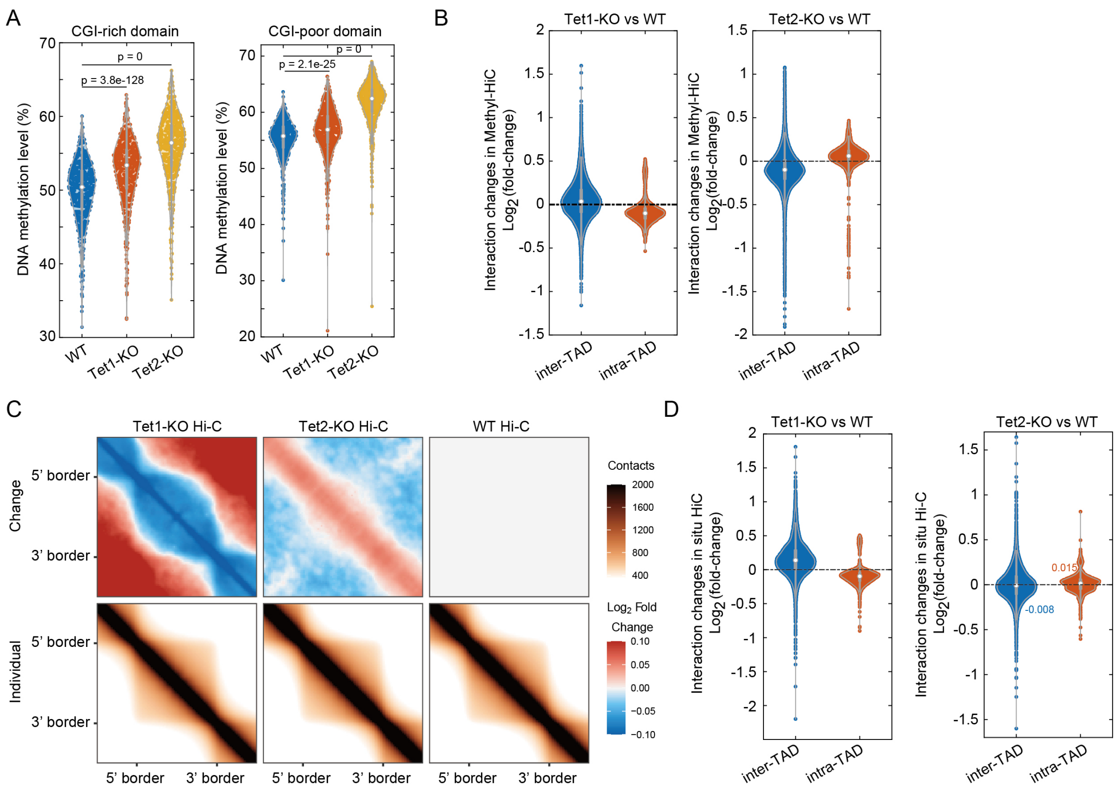

Figure S5. (**A**) DNA methylation distribution of CGI-rich and CGI-poor domains in WT, Tet1-KO and Tet2-KO samples. Welch's unequal variance t-test was performed. (**B**) Intra- and inter-TAD interaction variations (upon Tet1-KO and Tet2-KO) based on Methyl-HiC data. (**C**) ATA on WT, Tet1-KO and Tet2-KO samples. Hi-C data were used here. (**D**) Intra- and inter-TAD interaction variations (upon Tet1-KO and Tet2-KO) based on Hi-C.
